# Region-Specific Response of Astrocytes to Prion Infection

**DOI:** 10.3389/fnins.2019.01048

**Published:** 2019-10-09

**Authors:** Natallia Makarava, Jennifer Chen-Yu Chang, Rajesh Kushwaha, Ilia V. Baskakov

**Affiliations:** ^1^Center for Biomedical Engineering and Technology, University of Maryland School of Medicine, Baltimore, MD, United States; ^2^Department of Anatomy and Neurobiology, University of Maryland School of Medicine, Baltimore, MD, United States

**Keywords:** prion, prion diseases, astrocytes, microglia, reactive astrogliosis, chronic neuroinflammation, thalamus, hippocampus

## Abstract

Chronic neuroinflammation involves reactive microgliosis and astrogliosis, and is regarded as a common pathological hallmark of neurodegenerative diseases including Alzheimer’s, Parkinson’s, ALS and prion diseases. Reactive astrogliosis, routinely observed immunohistochemically as an increase in glial fibrillary acidic protein (GFAP) signal, is a well-documented feature of chronic neuroinflammation associated with neurodegenerative diseases. Recent studies on single-cell transcriptional profiling of a mouse brain revealed that, under normal conditions, several distinct subtypes of astrocytes with regionally specialized distribution exist. However, it remains unclear whether astrocytic response to pro-inflammatory pathological conditions is uniform across whole brain or is region-specific. The current study compares the response of microglia and astrocytes to prions in mice infected with 22L mouse-adapted prion strain. While the intensity of reactive microgliosis correlated well with the extent of PrP^Sc^ deposition, reactive astrogliosis displayed a different, region-specific pattern. In particular, the thalamus and stratum oriens of hippocampus, which are both affected by 22L prions, displayed strikingly different response of astrocytes to PrP^Sc^. Astrocytes in stratum oriens of hippocampus responded to accumulation of PrP^Sc^ with visible hypertrophy and increased GFAP, while in the thalamus, despite stronger PrP^Sc^ signal, the increase of GFAP was milder than in hippocampus, and the change in astrocyte morphology was less pronounced. The current study suggests that astrocyte response to prion infection is heterogeneous and, in part, defined by brain region. Moreover, the current work emphasizes the needs for elucidating region-specific changes in functional states of astrocytes and exploring the impact of these changes to chronic neurodegeneration.

## Introduction

Chronic neuroinflammation involves reactive microgliosis and astrogliosis, and is regarded as a common pathological hallmark of neurodegenerative diseases including Alzheimer’s, Parkinson’s, ALS and prion diseases (Perry et al., 2007; Schwartz et al., 2013; Streit et al., 2014; Gomez-Nicola and Perry, 2015). In prion diseases, the vast majority of previous studies addressing the role of neuroinflammation in chronic neurodegeneration focused on microglia (reviewed in Carroll and Chesebro, 2019). A number of studies showed that activation and proliferation of microglia occurs in the regions of prion or PrP^Sc^ accumulation and in response to PrP^Sc^ accumulation (Williams et al., 1997; Giese et al., 1998; Bate et al., 2002; Everbroeck et al., 2004; Puoti et al., 2005; Kercher et al., 2007; Sandberg et al., 2014; Greenlee et al., 2016; Vincenti et al., 2016). In prion diseases of animals and humans, activation and proliferation of microglia were found to occur at very early, subclinical stages (Baker and Manuelidis, 2003; Lu et al., 2004; Gomez-Nicola et al., 2013; Sandberg et al., 2014; Carroll et al., 2016). By the clinical onset of the disease, microglial populations expand as much as 10-fold (Gomez-Nicola and Perry, 2015). Over the years, evidence has been presented in favor of both a protective phenotype and inflammatory, neurotoxic phenotypes for microglia in the pathogenesis of prion diseases (Giese et al., 1998; Everbroeck et al., 2004; Kercher et al., 2007; Riemer et al., 2008; Kranich et al., 2010; Gomez-Nicola et al., 2013; Sakai et al., 2013; Grizenkova et al., 2014; Sorce et al., 2014; Muth et al., 2016; Zhu et al., 2016).

Considerably less is known about the role of reactive astrocytes. It has been well established that astrocytes can replicate and accumulate PrP^Sc^ independently of neurons (Raeber et al., 1997; Cronier et al., 2004; Aguzzi and Liu, 2017; Krejciova et al., 2017). Furthermore, expression of PrP^C^ on astrocytes was found to be sufficient for prion-induced chronic neurodegeneration (Jeffrey et al., 2004; Kercher et al., 2004). However, it remains to be shown how normal physiological functions of astrocytes change over the course of chronic neuroinflammation.

Astrocytes are responsible for a variety of physiological functions including maintenance of CNS homeostasis, modulation of neurotransmission, formation and elimination of synapses, regulation of blood flow, supplying energy and providing metabolic support to neurons, maintaining the blood–brain-barrier and more (Dallérac et al., 2018; Santello et al., 2019). In response to insults to the nervous system, astrocytes become activated and acquire reactive phenotypes that have been formally classified as A1 (pro-inflammatory or toxic), A2 (neurotrophic or neuroprotective) and PAN-reactive using terminology that parallels the M1 and M2 macrophage nomenclature (Ferrer, 2017; Liddelow and Barres, 2017). Such classification is excessively simplified. While researchers agree that populations of reactive astrocytes are heterogeneous, whether heterogeneity arises due to co-existing mixtures of A1 and A2 states, multiple distinct activation states in addition to classical A1 and A2 states, co-expression of markers of different activation states within individual cells, or all of the above, is not clear. Moreover, it is also not clear whether astrocytes in their reactive pro-inflammatory A1-like phenotypes lose their ability to perform normal physiological functions.

Recent studies on single-cell transcriptional profiling revealed seven molecularly distinct types of astrocytes with regionally specialized distribution in mouse CNS (excluding retina and spinal cord) under normal conditions (Zeisel et al., 2018). Moreover, localization of distinct types of astrocytes in separate domains was shown to be specified developmentally (Zeisel et al., 2018). Considerably less is known about functional specialization of astrocytes in different brain regions and their region-specific responses to chronic neurodegeneration. Recent work by Ben Barres and coworkers proposed that neuroinflammation of microglia drives astrocytes into pro-inflammatory reactive A1 states, which are toxic to neurons and oligodendrocytes (Liddelow and Barres, 2017; Liddelow et al., 2017). However, it remains unknown whether astrocytes respond to pro-inflammatory pathological conditions in a uniform pattern regardless of brain region.

The current study compared region-specific response of astrocytes to prion infection in mice infected with 22L mouse-adapted prion strain (Dickinson, 1976). We found that the intensity of reactive microgliosis correlated well with the extent of PrP^Sc^ deposition between the hippocampus and thalamus, whereas the intensity of astrogliosis did not. While PrP^Sc^ accumulates in both regions, stratum oriens of hippocampus and thalamus exhibit strikingly different morphology of reactive astrocytes and intensity of astrogliosis. The current study suggests that astrocyte response to prion infection is heterogeneous and, in part, defined by brain region.

## Materials And Methods

### Ethics Statement

This study was carried out in strict accordance with the recommendations in the Guide for the Care and Use of Laboratory Animals of the National Institutes of Health. The animal protocol was approved by the Institutional Animal Care and Use Committee of the University of Maryland, Baltimore (Assurance Number A32000-01; Permit Number: 0215002).

### Animals

C57BL/6J mice (females and males) were inoculated intracerebrally into the left hemisphere ∼2 mm to the left of the midline and ∼2 mm anterior to a line drawn between the ears with 20 μl of 1% 22L (*n* = 15 females, *n* = 10 males) or 10% mouse-adapted SSLOW (SSLOW-Mo, *n* = 16 females, *n* = 5 males) brain homogenates under isoflurane anesthesia. Inoculum is delivered slowly by a 26 G needle inserted to a depth of approximately 3 mm. All 22L and SSLOW-Mo-inoculated animals displayed signs of neurological disease consisting of hind-limb clasp and ataxia, and were unable to walk on a beam, developed kyphosis and became lethargic upon disease progression. Mice were considered terminally ill when they were unable to rear and/or lost 20% of their weight. At this point they were euthanized by CO_2_ asphyxia and decapitation. The disease status was confirmed by detecting PrP^Sc^ on Western blot in both 22L and SSLOW-Mo groups.

### Histopathological Study

Formalin-fixed brain halves divided at the midline (left hemisphere) were treated in formic acid (95%) to deactivate prion infectivity before being embedded in paraffin. 4 μm sections mounted on slides were processed for hematoxylin-eosin (H&E) staining and immunohistochemistry. To expose epitopes, slides were subjected to 20 min hydrated autoclaving at 121°C in trisodium citrate buffer, pH 6.0 with 0.05% Tween 20. For detection of disease-associated PrP, 5 min treatment with 88% formic acid was used following autoclaving. PrP was stained with anti-prion antibody SAF-84 (Cayman Chemical, Ann Arbor, MI). Rabbit anti-Iba1 (Wako, Richmond, VA) was used to stain microglia. Rabbit polyclonal anti-glial fibrillary acidic protein (GFAP) (Promega, Madison, WI), chicken polyclonal anti-GFAP (Sigma-Aldrich, St. Louis, MO), rabbit polyclonal anti-Aldh1l1 (Abcam, Cambridge, MA), and rabbit monoclonal anti-S100ß (Abcam, Cambridge, MA) were used to stain astrocytes. Detection was performed using DAB Quanto chromogen and substrate (VWR, Radnor, PA).

### Immunofluorescence and Quantification

For double immunofluorescence, chicken polyclonal anti-GFAP (Sigma-Aldrich, St. Louis, MO) antibody was used in combination with rabbit anti-Iba1 (Wako, Richmond, VA), rabbit polyclonal anti-Aldh1l1 (Abcam, Cambridge, MA), or rabbit monoclonal anti-S100ß (Abcam, Cambridge, MA). Secondary antibodies were goat anti-chicken AlexaFluor-488 and goat anti-rabbit AlexaFluor-546. Autofluorescence eliminator (Sigma-Aldrich, St. Louis, MO) was used according to the original protocol to reduce background fluorescence. Images were collected using an inverted microscope (Nikon Eclipse TE2000-U) equipped with an illumination system X-cite 120 (EXFO Photonics Solutions Inc., Exton, PA, United States) and a cooled 12-bit CoolSmap HQ CCD camera (Photometrics, Tucson, AZ, United States). Images were processed using WCIF ImageJ software (National Institute of Health, Bethesda, MD, United States).

Quantification of Iba1 and GFAP signals was performed using 4 animals per group. From each brain, 3 images of thalamus or stratum oriens of the hippocampus were collected under the ×20 objective. After subtraction of the background and threshold adjusting, particle measurements from at least ten 30 × 30 pixels ROIs surrounding microglia cells or encompassing astrocytic processes were collected from each image. Mean integrated densities were plotted. Error bars represent SEM.

Quantification of co-immunostaining for astrocytic markers was performed from the images collected under the ×60 objective. Background was subtracted, and the regions of nuclei defined by DAPI staining were removed from calculations because non-specific GFAP signal in normal thalamus was interfering with acquisition of low GFAP signal in astrocytes. For S100ß, 10 to 14 images were collected from 4 prion-infected and 4 normal brains, resulting in 40 to 44 images per brain region. For Aldh1l1, depending on the section quality, 3 to 12 images were collected from 4 prion-infected and 4 normal brains, resulting in 29 to 40 images per brain region. Mean integrated densities were plotted. Error bars represent SEM.

### qRT-PCR

Brains were divided at the midline, and right hemispheres were used to dissect hippocampi and thalami. 10% (wt/vol) homogenates were prepared within 1.5 ml tubes in Trizol (Thermo Fisher Scientific, Waltham, MA, United States), using RNase-free disposable pestles (Fisher Scientific, Hampton, NH). Total RNA was isolated by using Aurum Total RNA Mini Kit (Bio-Rad, Hercules, CA, United States) and subjected to DNase I digestion to remove contaminating genomic DNA. Total RNA was dissolved in elution buffer and stored at −80°C. An absorbance 260/280 value of ∼ 2.0, determined using NanoDrop ND-1000 Spectrophotometer (Thermo Fisher Scientific, Waltham, MA, United States), proved RNA purity. Reverse transcription was performed using 1 μg of extracted RNA and iScript cDNA Synthesis Kit (Bio-Rad, Hercules, CA, United States). qRT-PCR was performed in triplicate from three normal and three prion-infected animals using SsoAdvanced Universal SYBR Green Supermix (Bio-Rad, Hercules, CA, United States) using primer sequences for GFAP, S100ß, Aldh1l1, Iba1, MBP, NeuN, MAP2, Serping1, Ggta1, S100A10, TGM1 and Cxcl10 shown in Table 1. The PCR protocol consisted of 95°C for 2 min and 40 amplification cycles with the following steps: 95°C for 5 s, and 60°C for 30 s. The –ΔCt were quantified and analyzed by using CFX96 Touch Real-Time PCR Detection System (Bio-Rad, Hercules, CA, United States), and plotted by GraphPad Prism. SigmaPlot was used to present –ΔCt values in a 3D plot. The ΔC_t_ for each mouse RNA sample was calculated by subtracting the mean C_t_ of housekeeping gene GAPDH from the C_t_ of the gene of interest.

**TABLE 1:** Primer sequences for qRT-PCR.

**Primers**	**Accession number**	**Sequence**
GFAP	NM_001131020.1	F 5′-ACAGACTTTCTCCAACCTCCAG-3′ R 5′-CCTTCTGACACGGATTTGGT-3′
S100ß	NM_009115.3	F 5′-CTGGAGAAGGCCATGGTTGC-3′ R 5′-CTCCAGGAAGTGAGAGAGCT-3′
ALDH1L1	NM_027406.2	F 5′-CAGTTCTTCAAGGGGTCTGC-3′ R 5′-CAGAATTCGCATCCAAGAGC-3′
Iba1	NM_001361501.1	F 5′-GACGTTCAGCTACTCTGACTTT-3′ R 5′-GTTGGCCTCTTGTGTTCTTTG-3′
MBP	NM_001025251.2	F 5′-CTCAGAGGACAGTGATGTGTTT-3′ R 5′-CGCCTTGCCAGTTATTCTTTG-3′
MAP2	NM_001039934.1	F 5′-TGGTTCCAAGGATAACATCAAA-3′ R 5′-CATTTGGATGTCACATGGCTTA-3′
NeuN	NM_001039167.1	F 5′-ACCTACAGCATCGGAACCAT-3′ R 5′-TTGCTAGTAGGGGGTGAAGC-3′
Serping1	NM_009776.3	F 5′-TGATGGCGCCTTTCTTCTAC-3′ R 5′-CCACCTTGGCCTTCAAAGTA-3′
Ggta1	NM_001308300.1	F 5′-TCATCGGGTCCTACCTACAA-3′ R 5′-CTTCAGTCACCTGCTCCATAC-3′
Cxcl10	NM_021274.2	F 5′-AGTAACTGCCGAAGCAAGAA-3′ R 5′-GCACCTCCACATAGCTTACA-3′
Tgm1	NM_001161715.1	F 5′-GCCCTTGAGCTCCTCATTG-3′ R 5′-CCCTTACCCACTGGGATGAT-3′
S100a10	NM_009112.2	F 5′-GGGCTTCCAGAGCTTTCTATC-3′ R 5′-CTCCAGTTGGCCTACTTCTTC-3′
GAPDH	NM_001289726.1	F 5′-AACAGCAACTCCCACTCTTC-3′ R 5′-CCTGTTGCTGTAGCCGTATT-3′

*F- Forward, R- Reverse.*

### Data Availability

All data generated or analyzed during this study are included in this published article (and its Supplementary Information Files).

## Results

For examining region specific response, C57Bl/6J mice infected with 22L were euthanized at the advanced clinical stage of the disease (145–159 days postinoculation). PrP^Sc^ deposition, reactive microgliosis and astrogliosis were examined across whole brains using staining with anti-PrP antibody, Iba1 and GFAP, respectively. We found that among brain areas affected by 22L prions, two regions: thalamus and hippocampus, displayed the most drastic differences in response of astrocytes to PrP^Sc^ deposition (Supplementary Figure S1). For this reason, the current study will focus on detail comparison of reactive astrogliosis between these two regions.

**FIGURE 1:**
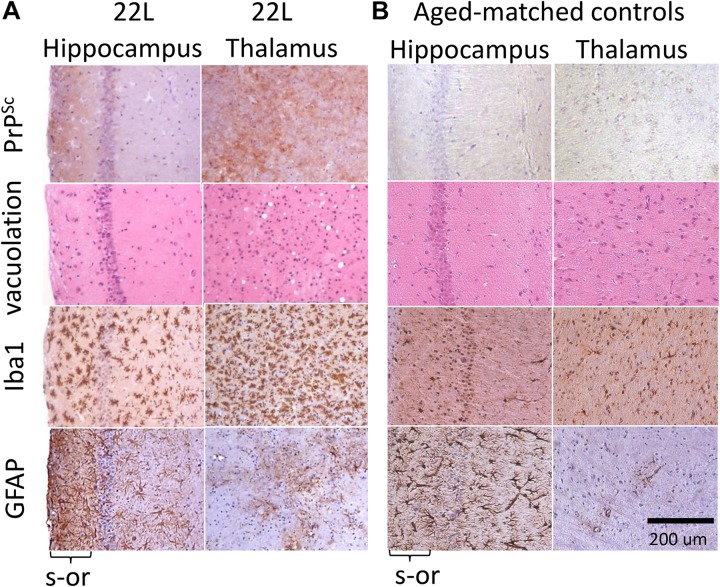
Histopathological analysis of 22L-infected mouse brains. **(A)** Deposition of PrP^Sc^ stained with SAF-84 antibody, vacuolation stained with hematoxylin-eosin, and activated microglia and astrocytes stained for Iba1 and GFAP, respectively, in hippocampus and thalamus of C57Bl/6J mice inoculated with 22L prions (s-or, stratum oriens). **(B)** Staining of normal age-matched control mice is shown as reference. Scale bar = 200 μm.

**FIGURE 2:**
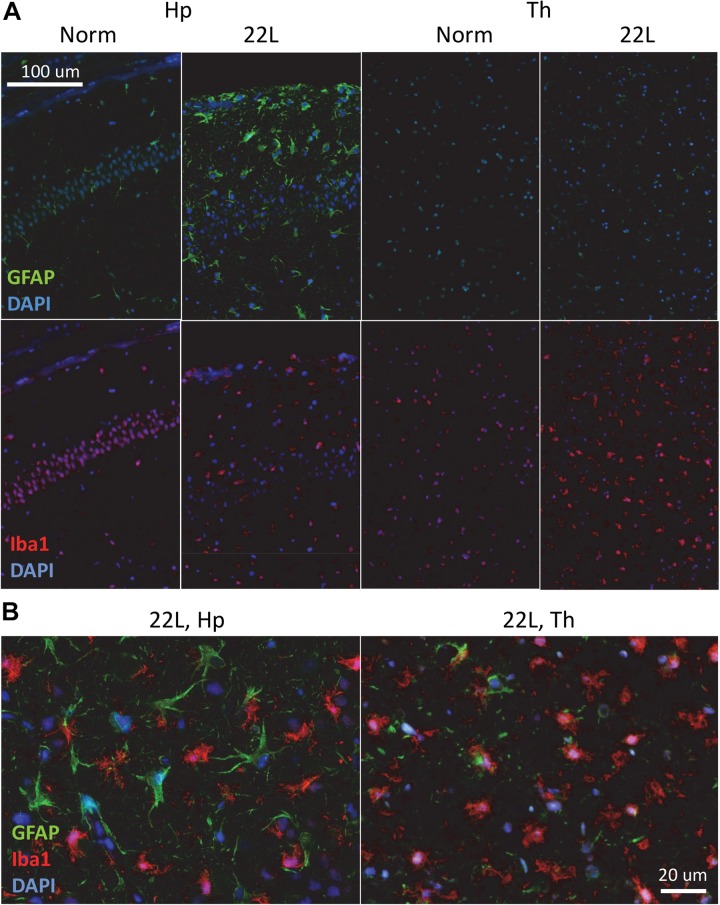
Co-immunostaining of microglia and astrocytes. **(A)** Representative images of fluorescent co-immunostaining of microglia (Iba1, red) and astrocytes (GFAP, green) in thalamus (Th) and hippocampus (Hp) of C57Bl/6J mice inoculated with 22L prions and normal age-matched controls (Norm). DAPI (blue) was used for staining of nuclei. **(B)** Magnified merged images of 22L-infected stratum oriens of the hippocampus (left) and thalamus (right).

Immunostaining using anti-PrP antibody revealed strong deposition of PrP^Sc^ in thalamus, and milder PrP^Sc^ deposition in hippocampus (Figures 1A,B). Thalamus also had pronounces vacuolation visible with hematoxylin-eosin staining. As judged from Iba1 staining, which is indicative of microglia inflammation and proliferation, the pattern of reactive microgliosis in 22L infected brains resembled the pattern of PrP^Sc^ deposition with respect of spatial distribution and intensity (Supplementary Figure S1). In particular, thalamus had more inflamed microglia cells than hippocampus. Increased GFAP staining, a marker of astrocyte activation, was also observed in conjunction with PrP^Sc^ deposition and microgliosis (Supplementary Figure S1). However, contrary to the intensity of microglia and PrP^Sc^ staining, the intensity of GFAP staining was much weaker in thalamus relative to stratum oriens of hippocampus (Figure 1A and Supplementary Figure S1). In a similar manner, substantial region-specific differences in response to prion infection were also evident from co-immunostaining of microglia (Iba1, red) and astrocytes (GFAP, green) (Figure 2 and Supplementary Figure S2). Again, astrocytes in hippocampus and cortex had hypertrophic cell bodies and processes compared to control (Figure 2 and Supplementary Figure S2). In thalamus, proliferating and/or migrating reactive microglia were the most notable features, whereas astrocytes appeared significantly less hypertrophic than in hippocampus (Figure 2).

**FIGURE 3:**
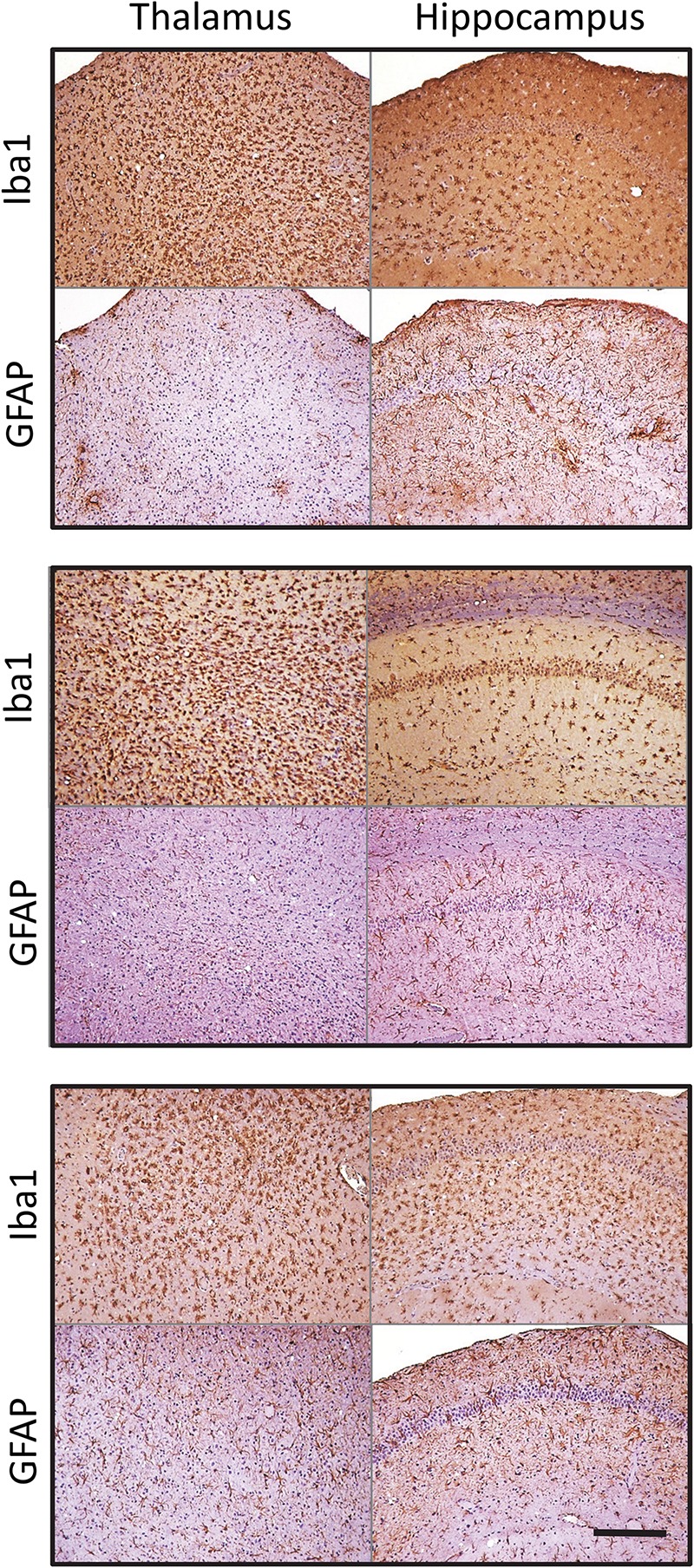
Microglia and astrocyte reaction in the thalamus and hippocampus of SSLOW-Mo-infected mice. Activated microglia and astrocytes stained for Iba1 and GFAP, respectively, in hippocampus and thalamus of C57Bl/6J mice inoculated with SSLOW-Mo. Three panels represent three individual animals. Scale bar = 200 μm.

Similar phenomenon was also observed for a mouse-adapted SSLOW (SSLOW-Mo), a strain of synthetic origin (Figure 3 and Supplementary Figure S3) (Makarava et al., 2010, 2012). In a manner similar to 22L animals, in animals infected with SSLOW-Mo the intensity of reactive microgliosis correlated well with the extent of PrP^Sc^ deposition on regional and sub-regional levels (Supplementary Figure S3). This was not the case for reactive astrogliosis. Again, SSLOW-Mo animals displayed highly enlarged hypertrophic astrocytes in the cortex and sub-regions of hippocampus, whereas in the thalamus the change of astrocytic morphology remained very mild, which contrasted with massive presence of reactive microglia within the same areas (Figure 3 and Supplementary Figure S3). The fact that the same phenomenon is observed for strains of natural and synthetic origin suggests that the response of astrocyte to prion infection appears to be defined by a brain area.

**FIGURE 4:**
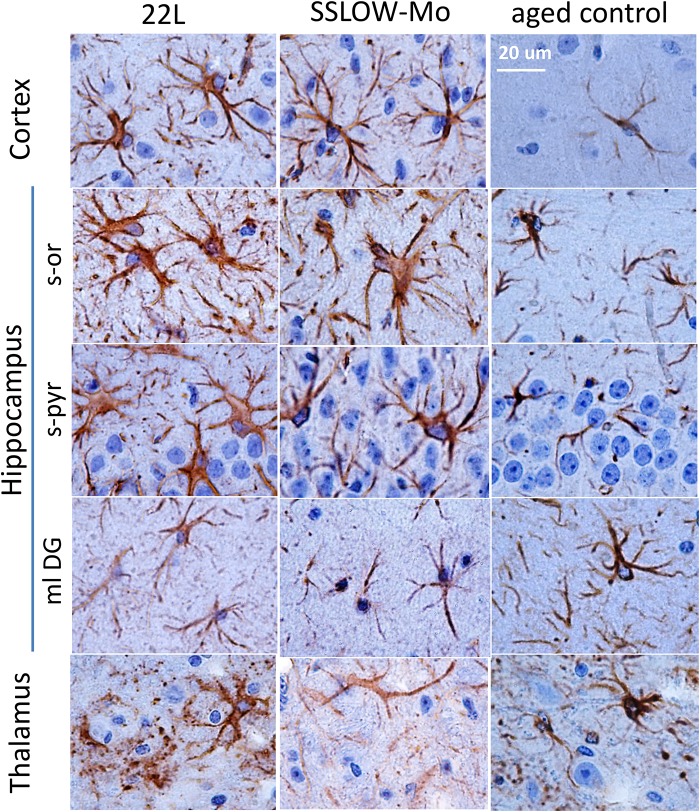
Analysis of region-specific astrocyte morphology. Imaging of astrocytes stained for GFAP in cortex, hippocampus (s-or, stratum oriens; s-pyr, stratum pyramidale; ml DG, molecular layer of dentate gyrus) and thalamus of C57Bl/6J mice inoculated with 22L prions, SSLOW-Mo and normal aged controls. Scale bar = 20 μm.

Magnified images of brain sections stained with GFAP revealed regional and sub-regional differences in astrocyte morphology (Figure 4). In both 22L and SSLOW-Mo animals, the molecular layer of dentate gyrus (ml DG) of hippocampus did not exhibit drastic changes in the shape of astrocytes relative to the age-matched controls, whereas the stratum pyramidale (s-pyr) and stratum oriens (s-or) of hippocampus as well as cortex contained hypertrophic astrocytes characterized by enlarged cell bodies with prominent nuclei and thick processes (Figure 4). In contrast to enlarged hypertrophic astrocytes of hippocampus and cortex, astrocytes of the thalamus showed strikingly different morphologies in both 22L and SSLOW-Mo animals (Figure 4). Such morphologies resembled beaded or fragmented processes and puncta-like GFAP-positive structures interpreted by previous studies as signatures of degenerating astrocytes (Martin et al., 2001; Hsu et al., 2018).

**FIGURE 5:**
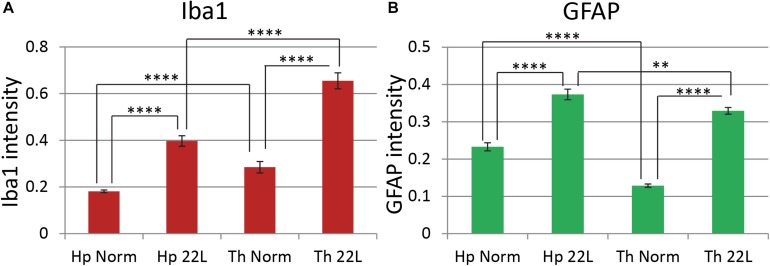
Comparison of region-specific microglial and astrocytic response to 22L prion infection. Quantification of Iba1 **(A**, red) and GFAP **(B**, green) immunofluorescence in thalamus (Th) and stratum oriens of the hippocampus (Hp) of C57Bl/6J mice inoculated with 22L prions and normal age-matched controls (Norm). The data (n = 4 animals per group) were collected and plotted as mean integrated density ± Standard Error of measurement. Statistical significance (p) was calculated by Student’s t-test and indicated as ^∗∗^ for *p* < 0.01 and ^****^ for *p* < 0.0001.

Quantitative comparison of Iba1 and GFAP immunofluorescence in the hippocampus and the thalamus confirmed that in 22L-infected animals reactive microgliosis was more intense in the thalamus than the hippocampus, whereas astrogliosis was more intense in the hippocampus than the thalamus (Figures 5A,B). In part, the weaker astrocytic response in thalamus could be due to region-specific functional heterogeneity of astrocytes in normal brains, as suggested by lower levels of GFAP expression by thalamic astrocytes relative to hippocampal astrocytes in normal animals (Figure 5B). Variations in Iba1 and GFAP signals in normal aged mice, as well as a different degree of microglial and astrocytic responses to the prion infection were observed in individual animals (Supplementary Figure S4). Nevertheless, in hippocampi of normal animals astrocytes consistently displayed 1.6–2.2 fold higher GFAP signal relative to the normal thalami (Supplementary Figure S4). In 22L-infected animals, GFAP intensities were of similar values or lower in the thalami relative to the hippocampi. Microglia in 22L-infected thalami consistently showed higher intensity of Iba1 staining relative to the hippocampi (Supplementary Figure S4). This data suggests that, indeed, region-specific differences between astrocytes exist, and that these differences might contribute to the region-specific response of astrocytes to prion infection.

**FIGURE 6:**
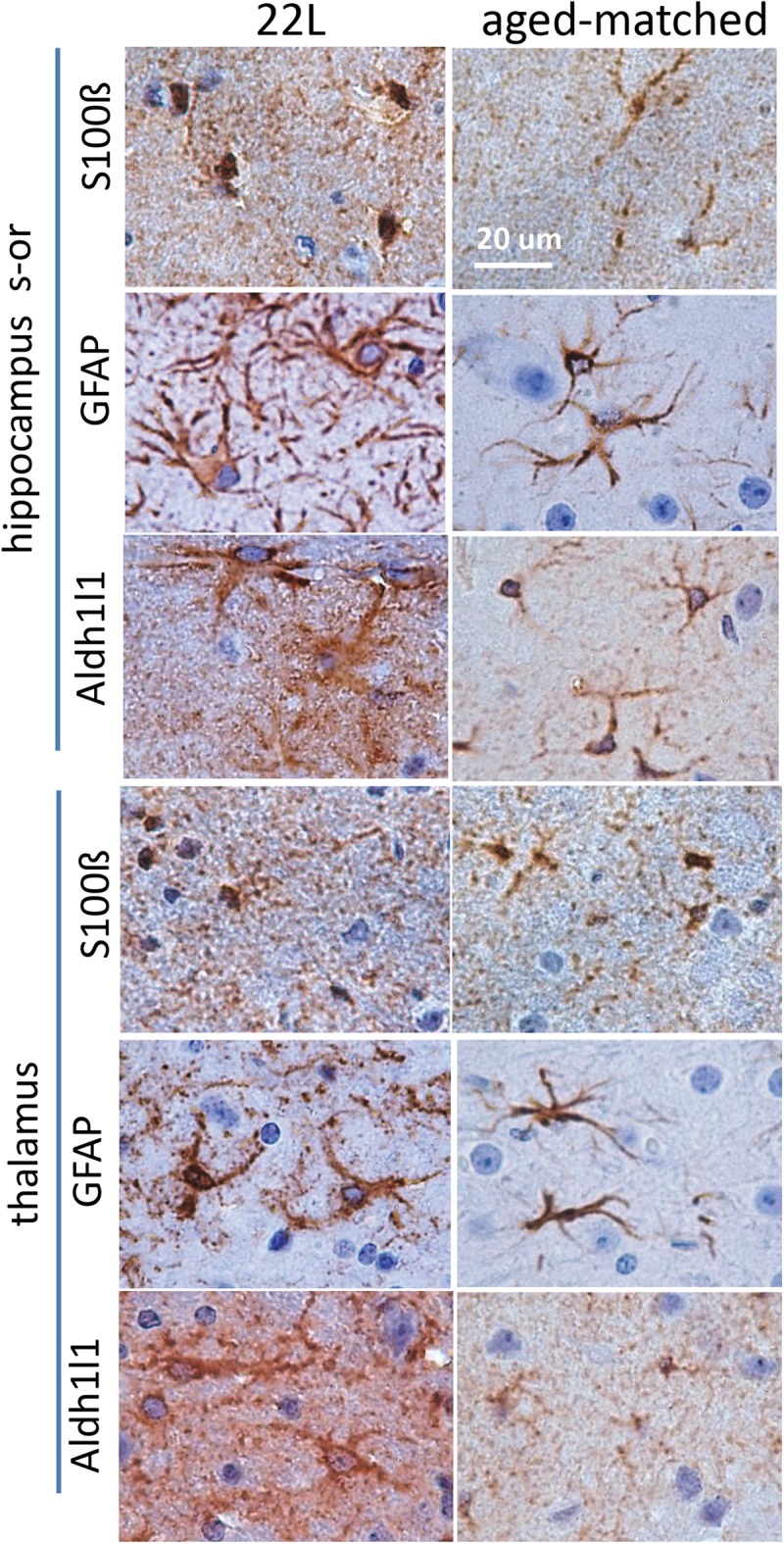
Analysis of region-specific astrocyte morphology. Imaging of astrocytes stained for S100ß, GFAP or Aldh1l1 in stratum oriens of hippocampus (s-or) and thalamus of C57Bl/6J mice inoculated with 22L prions and normal age-matched controls. Scale bar = 20 μm.

**FIGURE 7:**
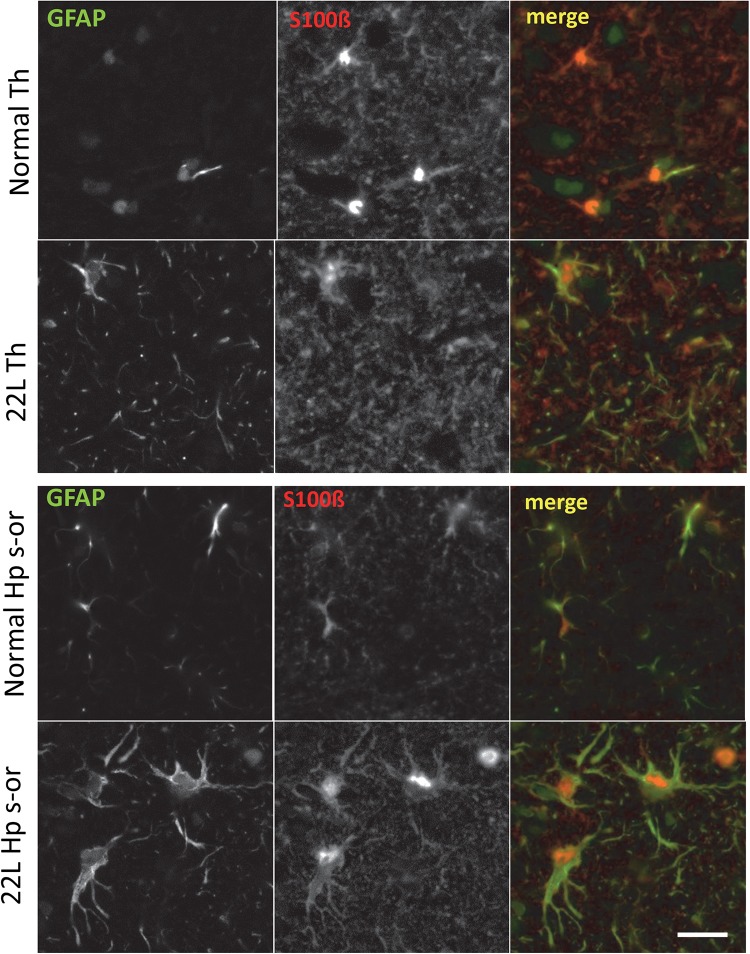
Co-immunostaining for GFAP and S100ß. Representative images of fluorescent co-immunostaining for GFAP (green) and S100ß (red) in thalamus (Th) and stratum oriens of the hippocampus (Hp s-or) of C57Bl/6J mice inoculated with 22L prions and normal age-matched controls (Norm). Scale bar = 10 μm.

**FIGURE 8:**
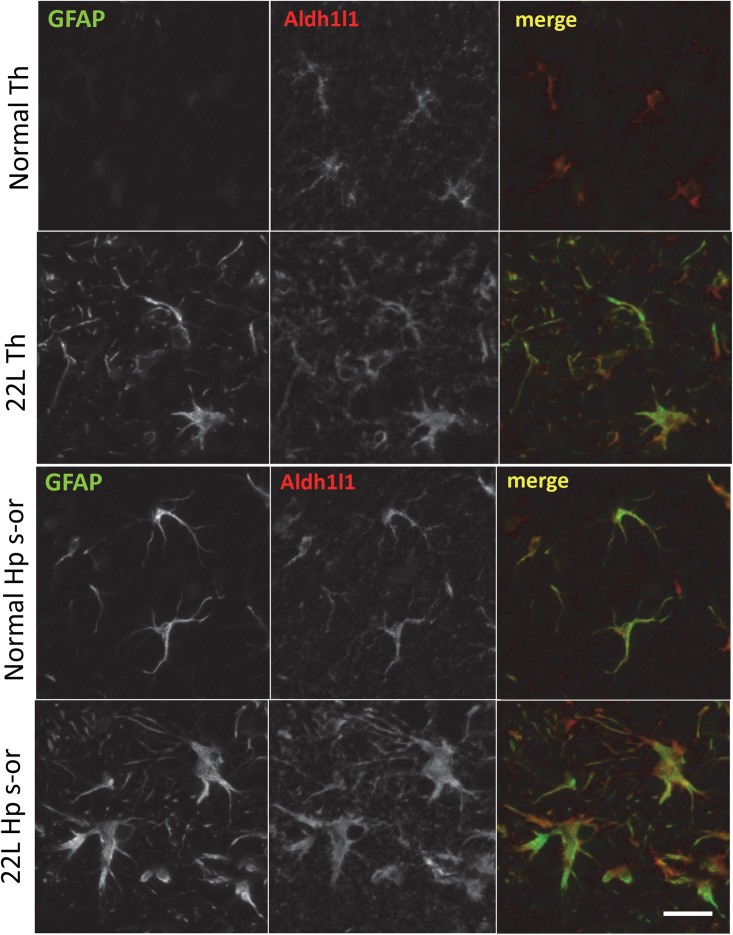
Co-immunostaining for GFAP and Aldh1l1. Representative images of fluorescent co-immunostaining for GFAP (green) and Aldh1l1 (red) in thalamus (Th) and stratum oriens of the hippocampus (Hp s-or) of C57Bl/6J mice inoculated with 22L prions and normal age-matched controls (Norm). Scale bar = 10 μm.

**FIGURE 9:**
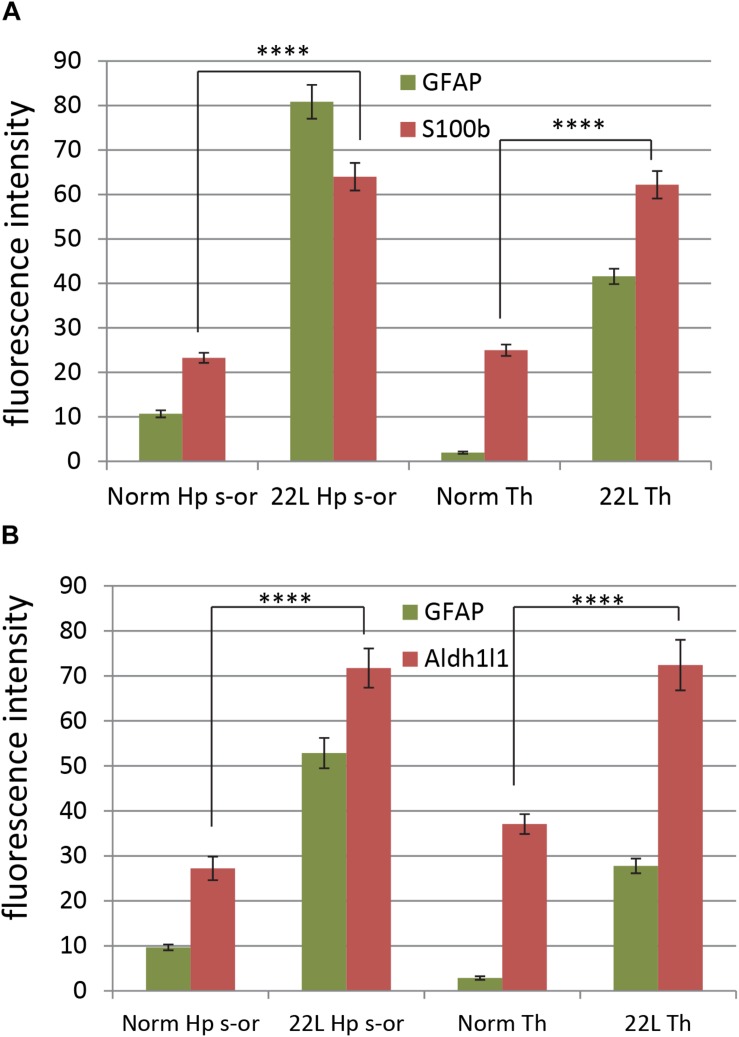
Quantification of astrocyte-specific markers S100ß and Aldh1l1. Quantification of immunofluorescence for astrocyte-specific markers S100ß **(A)** and Aldh1l1 **(B)** in thalamus (Th) and stratum oriens of the hippocampus (Hp s-or) of C57Bl/6J mice inoculated with 22L prions and normal age-matched controls (Norm). Quantification of GFAP is provided as a reference. The data (n = 4 animals per group) were collected and plotted as mean integrated density ± Standard Error of measurement. Statistical significance (p) was calculated by Student’s t-test and indicated as ^****^ for *p* < 0.0001.

To probe region-specific phenotypic differences between astrocytes further, two additional astrocytic markers were selected, S100 calcium binding protein B (S100ß) and aldehyde dehydrogenase 1 family member L1 (Aldh1l1) (Cahoy et al., 2008). Consistent with the results of GFAP staining, both S100ß and Aldh1l1 confirmed that astrocytes in the hippocampi of 22L animals had substantially enlarged cell bodies and processes in comparison to the thalami or the normal controls (Figures 6–8). In 22L animals, the changes in thalamic astrocytes were considerably more modest than in hippocampus (Figure 6). Detailed analysis of fluorescence immunohistochemistry images revealed region-specific differences in subcellular localization of astrocytic markers. In hippocampi of 22L animals, S100ß exhibited very intense nuclear stating, whereas in thalami S100ß showed a more equal distribution between nuclei and peripheral processes (Figures 6, 7). This pattern resembled cellular localization of S100ß in the thalami of normal controls (Figures 6, 7). In a cell, GFAP primarily localizes in the thick main processes, whereas Aldh1L1-staining usually reveals the highly branched astrocyte cell morphology including the cell body and its extensive processes. Regardless of the disease status or brain region, Aldh1l1 was found in cell bodies and along peripheral processes (Figures 6, 8). However, in the hippocampi of 22L animals the intensity of Aldh1l1 in peripheral processes was stronger relative to the astrocytic processes in the thalami (Figure 8). Quantitative comparison of S100ß and Aldh1l1 immunofluorescence in cell processes and soma (nuclei were excluded from the calculations due to non-specific signal) confirmed that the intensity of both markers elevated in both regions as a result of prion infection (Figures 9A,B). However, while GFAP showed lower fluorescence intensity in 22L-infected thalami than hippocampi, the integrated densities of S100ß and Aldh1l1 were similar in the two regions (Figures 9A,B), suggesting that in prion infected brains thalamic astrocytes differ from hippocampal astrocytes in GFAP, but not in Aldh1l1 or S100ß.

**FIGURE 10:**
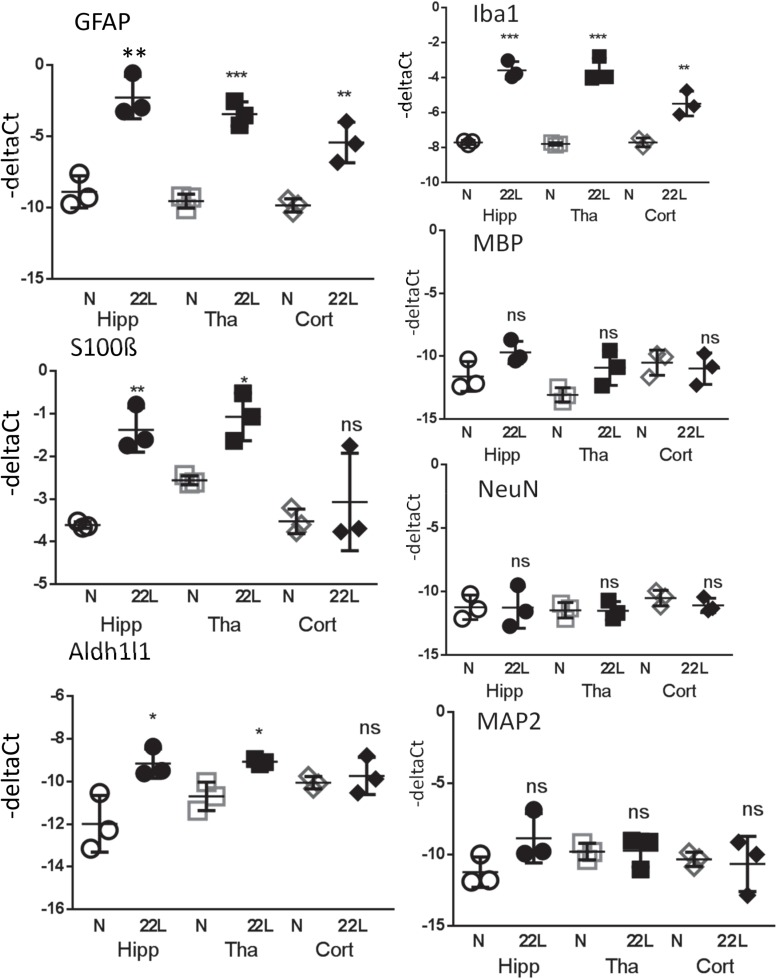
Analysis of gene expression by qRT-PCR. The ΔC_t_ of astrocytic markers GFAP, S100ß and Aldh1l1, microglial marker Iba1, oligodendrocytic marker MBP and neuronal markers NeuN and MAP2 in thalamus (Tha), hippocampus (Hipp) and cortex (Cort) of C57Bl/6J mice inoculated with 22L prions and normal age-matched controls (N). The mean and standard deviation are shown (n = 3 individual animals). Each symbol represents an individual mouse. GAPDH was used as a housekeeping gene. Statistical significance (p) was calculated by Student’s unpaired t-test and indicated as ^∗^ for *p* < 0.05; ^∗∗^ for *p* < 0.01; ^∗∗∗^ for *p* < 0.001; ns for non-significant.

For testing whether region-specific response could be attributed to differences in gene expression, qRT-PCR analysis of astrocytic and non-astrocytic markers in normal and 22L-infected brains was performed. A statistically significant increase in expression of S100ß gene was found in thalami and hippocampi in 22L-infected animals relative to the normal controls (Figure 10). Aldh1l1 expression pattern was also consistent with immunofluorescence data, although the increase in Aldh1l1 expression in 22L brains was at a borderline of statistical significance (Figure 10). In cortex, expression of S100ß and Aldh1l1 in 22L animals did not show statistically significant differences relative to normal controls (Figure 10). While elevated levels of GFAP in response to prion infection were consistent with immunofluorescence data, regional differences in GFAP expression were not evident from qRT-PCR (Figure 10). Analysis of immunofluorescence and gene expression together suggests that the phenotypes of reactive astrocytes are regulated via multiple mechanisms and not only by gene expression.

For testing whether an increase in gene expression of astrocyte-specific genes could be attributed to changes in cell composition and, specifically, neuronal death, qRT-PCR analysis of neuronal and oligodendrocytic markers was performed (Figure 10). Despite the advanced stage of the disease and pronounced neuroinflammation, the changes in oligodendrocytic (MBP) and neuronal markers (NeuN, MAP2) in hippocampus, thalamus and cortex relative to the normal controls were found to be very minor, if any (Figure 10). These results supports previous studies and illustrate that neuroinflammation in prion-infected brains precedes neuronal loss (Baker and Manuelidis, 2003; Lu et al., 2004; Gomez-Nicola et al., 2013; Carroll et al., 2016), and that the neuronal loss is minor even at the advanced stages of the disease (Majer et al., 2012, 2019). Consistent with the results on immunostaining of brain sections, expression of Iba1 was upregulated in all three regions (Figure 10). These results also suggest that the observed increase in expression of astrocyte-specific markers could not be attributed to changes in cell composition in prion-infected brains.

To probe phenotypic changes in astrocytes further, qRT-PCR analysis of A1 markers (Serping1, Ggta1), A2 markers (Tgm1, S100a10) and Cxcl10, a pro-inflammatory cytokine, was conducted. Consistent with previous studies (Carroll et al., 2016; Carroll and Chesebro, 2019), pro-inflammatory cytokine Cxcl10 was upregulated in 22L animals relative to normal controls in all three regions analyzed (Supplementary Figure S5). A statistically significant increase in expression of both A2 markers was observed in thalamus and hippocampus (Supplementary Figure S5). The hippocampus and cortex, but not thalamus, showed statistically significant increase in expression of A1 markers (Supplementary Figure S5). Together, these results suggest that region-specific differences in astrocyte response to prion infection do exist.

## Discussion

Elucidating region-specific diversity of astrocytes is important for better understanding of the role the neuroinflammation plays in chronic neurodegeneration. Reactive astrogliosis, routinely observed immunohistochemically as an increase in GFAP signal, is one of the central features of prion and other neurodegenerative diseases (Kovacs et al., 2013; Jeffrey et al., 2014; Katorcha et al., 2018). Because accumulation of PrP^Sc^ in 22L-infected animals is mainly associated with astroglia regardless of brain area (Carroll et al., 2016), we expected that the degree of astrogliosis monitored by GFAP staining would correlate with the intensity of PrP^Sc^ deposition. Both immunohistochemistry and qRT-PCR showed an increase in astrocytic and microglial markers in mouse brains infected with prions. However, while the intensity of reactive microgliosis correlated well with PrP^Sc^ deposition between hippocampus and thalamus, the intensity of astrogliosis evident by GFAP immunostaining did not. Immunostaining for astrocyte-specific markers revealed differences in patterns of expression between 22L-infected hippocampus and thalamus supporting the hypothesis that the response of astrocytes to prion infection is determined in part by brain region. In the thalamus, prion infection leads to a massive microglia proliferation and activation, whereas the response of astrocytes was muted. Very mild repose of astrocytes in thalamus contrasted with their reaction in stratum oriens of hippocampus. Importantly, we observed this phenomenon not only for 22L, but also for SSLOW-Mo, a strain of synthetic origin (Figures 3, 4 and Supplementary Figure S3). Similar to 22L, animals infected with SSLOW-Mo displayed hypertrophic astrocytes in the cortex and some regions of hippocampus, while the change of astrocytic morphology in the thalamus remained mild.

One can attribute mild increase in GFAP staining in thalamus relative to hippocampus or cortex (Figure 1 and Supplementary Figure S1) to differences in the kinetics of PrP^Sc^ accumulation in different brain regions and the possibility that the thalamus is affected only at the later stages of the disease relative to other brain areas. However, such possibility contradicts the experimental data. Previous studies that employed several mouse adapted strains including 22L (Sandberg et al., 2014; Carroll et al., 2016), as well as our unpublished observations, indicate that the thalamus is impaired at the early stages and is most severely affected by the advanced stage of the disease. Indeed, thalamus was found to be the most severely affected with respect to PrP^Sc^ deposition and reactive microgliosis in the current study (Figures 1, 2 and Supplementary Figure S1). Yet, GFAP signal in 22L-infected thalamus, although elevated in comparison with age-matched normal thalamus, was lower than in the hippocampus. Staining with other markers of astrocytes (Aldh1l1 and S100ß) ruled out a delay in the activation of astrocytes in the thalamus. Moreover, the fact that in 22L animals Aldh1l1 and S100β signals were elevated to a similar extent in the thalamus and in the hippocampus argues against astrocyte degeneration in the thalamus (Figure 9) Together, our data suggest that astrocytes are activated in thalamus, hippocampus and cortex, yet display different region-specific response to prions. We speculate that region-specific phenotypic differences in normal astrocytes might have resulted in their different response to the disease.

In the current study, the degree of astrocytic changes depended on a detection technique and a chosen marker. While analysis of gene expression often is a method of choice for elucidating functional diversity, unless performed for a single cell, this method lacks the ability to focus on individual cells in defined small regions of interest. Instead, it provides an insight into gene expression averaged across cell population in a specified brain region. Bulk analysis of astrocytic markers by qRT-PCR revealed reactive astrogliosis, yet failed to detect fine differences within individual brain regions in prion-infected mice (Figure 10). Nevertheless, fluorescent double-immunostaining allowed us to focus on a cell-specific analysis in particular areas revealing more informative patterns of neuroinflammation.

Glial fibrillary acidic protein belongs to a family of intermediate filament proteins that serve as a component of the cytoskeleton in astrocytes. In addition to its architectural function, recent studies revealed that the level of GFAP expression modulates astrocytic release of pro-inflammatory cytokines including Cxcl10 (Kramann et al., 2019). The expression of GFAP was found to be essential for reactive astrogliosis and glial scar formation (Pekny and Pekna, 2004). S100ß is Ca^2+^-binding protein of the EF-hand type that is localized in the cytoplasm and nucleus, and could also be secreted. S100ß is implicated in diverse cellular functions including regulation of astrocyte shape, migration and modulating long-term neuronal synaptic plasticity (Nishiyama et al., 2002; Brozzi et al., 2009). At low, nanomolar concentrations, S100ß was found to act as a neurotrophic factor promoting neuronal survival, whereas at high, micromolar concentrations, S100ß caused neuronal apoptosis by direct action on neurons and via activation of microglia (Bianchi et al., 2007; Sorci et al., 2010). Morphological differences between astrocytes in hippocampus and thalamus with respect to intensity and subcellular staining patterns of GFAP and S100β are consistent with our hypothesis that the phenotypic region-specific differences in normal astrocytes contribute to the differential response of astrocytes to prion infection. Nevertheless, this topic warrants further investigation using more comprehensive approaches. Overall, the current study suggests that the degree of astrocytic changes under chronic neuroinflammation reflects initial regional heterogeneity of astrocytes in a normal brain, as well as their differential response.

**FIGURE 11:**
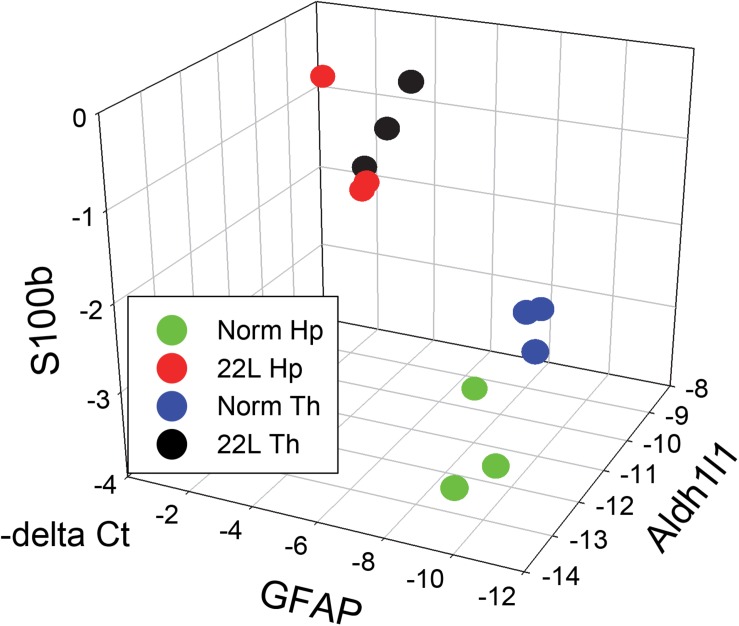
3D analysis of astrocytic phenotype. ΔC_t_ data of the three astrocytic markers GFAP, S100ß and Aldh1l1 in thalamus (Th) and hippocampus (Hp) of C57Bl/6J mice infected with 22L prions and normal age-matched controls are plotted in a 3D format. Each symbol represents an individual mouse. In normal mice, the pattern of gene expression shows two clusters, thalamus- and hippocampus-specific (Th – blue and Hp – green, respectively), which merge into one cluster (Th – black and Hp – red) in prion-infected mice.

The results of the current study also suggest that region specificity that astrocytes exhibit in a normal brain might diminish with prion infection (Figure 11). The degree of the differences with respect to gene expression between animal groups and within each group can be visualized by presenting the data on astrocytic markers in a 3D plot. qRT-PCR suggested that with the prion disease, the astrocytes undergo a considerable change. The region-specific differences in gene expression observed in normal control brains appear to be diminished with prion infection (Figure 11). Since the current study is limited to the analysis of astrocyte response at the advanced stage of the disease, the information captured may not represent the actual role of astrogliosis during early pathogenesis of prion disease. In future studies, it would be interesting to analyze more astrocytic genes using transcriptomic methods at different stages of the disease to gain insight into a region-specific astrocytic response.

The vast majority of previous studies on neuroinflammation in prion diseases focused on elucidating the role of microglia (reviewed in Carroll and Chesebro, 2019). Considerably less in know about contribution of astrocytes and their role in chronic neurodegeneration (reviewed in Ferrer, 2017). Under normal conditions, astrocytes perform a number of physiological functions essential for neuronal function and CNS homeostasis (Dallérac et al., 2018; Santello et al., 2019). It is not clear if any of the normal functions of astrocytes are undermined under chronic neuroinflammation and neurodegeneration. Moreover, the question of whether under chronic neurodegeneration astrocytes acquire neurotoxic phenotype or, on the contrary, boost their neuroprotective potential is under intense debate. On one hand, the potentially protective role of astrocytes in prion diseases is pointed out by the work illustrating the ability of astrocytes cultured *in vitro* to uptake and degrade PrP^Sc^ (Choi et al., 2014). Moreover, upregulation of astrocyte-specific proteins involved in protection against protein misfolding, glutamate excitotoxicity and oxidative stress was found in prion-infected mice at the terminal stages of the disease (Asuni et al., 2014). On the other hand, under chronic neurodegenerative conditions including prion diseases, astrocytes are primed to produce an exaggerated pro-inflammatory response (Hennessy et al., 2015). Like microglia in the M1 state, in the pro-inflammatory A1 state, astrocytes lose the ability to promote neuronal survival and, instead, induce death of neurons and oligodendrocytes (Liddelow et al., 2017). Furthermore, there is evidence suggesting that reactive A1 astrocytes lose their normal physiological functions important for supporting neurons (Clarke et al., 2018).

According to a recent hypothesis introduced by Ben Barres, inflammation of microglia results in the release of astrocyte-activating signals that drive astrocytes into pro-inflammatory A1 reactive states causing to release neurotoxins, which induce apoptosis of neurons and oligodendrocytes (Liddelow and Barres, 2017; Liddelow et al., 2017). In support of this hypothesis, blocking of reactive microglia stimuli that induce A1 phenotype in astrocytes was found to be neuroprotective under chronic neurodegeneration conditions (Yun et al., 2018). Moreover, consistent with this hypothesis, previous studies demonstrated attenuation of astrocytic gliosis and a delay of clinical onset of prion diseases in mice deficient of interleukin-1 receptor, which is activated by proinflammatory cytokines produced by microglia (Schultz et al., 2004). While the general concept introduced by Barres is useful for establishing causative relationships between activation of microglia and astrocytes and neuronal death, a number of potential challenges have to be considered for advancing this hypothesis further. First, expression of genes associated with neuroinflammation and synapse elimination was found to be elevated in astrocytes even in normal aging (Soreq et al., 2017; Boisvert et al., 2018; Clarke et al., 2018). How pro-inflammatory changes associated with normal aging affect the ability of astrocytes to respond to reactive microglia under chronic neuroinflammation remains to be elucidated. Second, diversity of functional states of astrocytes and the differential susceptibility of functionally diverse astrocyte populations to pro-inflammatory stimuli have to be considered (Clarke et al., 2018). Third, according to recent studies, the rates of astrocyte aging under normal conditions vary as a function of brain region (Boisvert et al., 2018). Whether the region-specific differences in aging impact susceptibility of these regions to chronic neurodegeneration remains to be addressed.

The current study suggests that the relationship between reactive microglia and astrocytes appears to be more complex than one could propose based on the Barres’s hypothesis. In the current study, the pro-inflammatory marker (Cxcl10) was found to be upregulated to the same extent in all three brain regions analyzed in 22L animals relative to the controls, while the markers of A1 and A2 showed region-specific nuances in their differential expression. Hippocampus displayed significant increase in expression of A1 and A2 markers, whereas in thalamus only A2 markers were upregulated with statistical significance. Cortex showed a more complex pattern of upregulation (Supplementary Figure S5). These results might suggest a reason why astrocytes in different brain regions react differently to prion infection. While A1, A2, and PAN markers have been employed for assessing reactive phenotypes of astrocytes, most of the A1, A2 or PAN biomarkers are not expressed by astrocytes but by other cell types (microglia, endothelium, neurons, oligodendrocytes). Therefore, analysis of their expression might not be informative for describing intrinsic phenotypic differences between astrocytes *per se*, but instead could be useful for reporting changes in region-specific micro-environments that direct astrocytic response under disease conditions. Nevertheless, while both the thalamus and stratum oriens of the hippocampus exhibited strong microgliosis, only stratum oriens displayed a prominent hypertrophy of astrocytes, whereas microgliosis in the thalamus coexisted with only a modest increase in GFAP signal. Further studies on the regional activation potential of the astrocytes is important for understanding why certain brain regions are affected by neurodegeneration, and whether astrocytes are helpful or detrimental players in the course of the disease.

## Data Availability Statement

All datasets generated for this study are included in the manuscript/Supplementary Files.

## Ethics Statement

The animal study was reviewed and approved by the Institutional Animal Care and Use Committee of the University of Maryland, Baltimore (Assurance Number A32000-01; Permit Number: 0215002).

## Author Contributions

IB and NM conceived the idea, designed the experiments, and wrote the manuscript. RK designed and tested primers for qRT-PCR. JC and NM performed the experiments and analyzed the data. All authors read and approved the final manuscript.

## Conflict of Interest

The authors declare that the research was conducted in the absence of any commercial or financial relationships that could be construed as a potential conflict of interest.
