# Supplementary material for: Region-Specific Response of Astrocytes to Prion Infection
Source: Front Neurosci. 2019 Oct 9;13:1048. doi: 10.3389/fnins.2019.01048 (PMC6794343; doi:10.3389/fnins.2019.01048)
Supplement: Supplementary file 1 [file Data_Sheet_1.PDF]

Supplementary Information

Region-specific response of astrocytes to prion infection

Natallia Makarava[1,2], Jennifer Chen-Yu Chang[1,2], Rajesh Kushwaha[1,2], Ilia V. Baskakov[1,2#]

[1] Center for Biomedical Engineering and Technology, University of Maryland School of Medicine, Baltimore, MD, 21201, United States of America; [2]Department of Anatomy and Neurobiology, University of Maryland School of Medicine, Baltimore, MD, 21201, United States of America

[#] To whom correspondence should be addressed: Center for Biomedical Engineering and Technology, University of Maryland School of Medicine, Baltimore, 111 S. Penn St., Baltimore, MD 21201. Phone: 410-706-4562; FAX: 410-706-8184. Email: Baskakov@som.umaryland.edu

Supplementary Figure Legends

Figure S1. Histopathological analysis of 22L-infected mouse brains. Low magnification images of deposition of PrP^{Sc} stained with SAF-84 antibody, and activated microglia and astrocytes stained for Iba1 and GFAP, respectively, in cortex, hippocampus and thalamus of C57Bl/6J mice inoculated with 22L prions. Scale bar = 500 μm.

Figure S2. Co-immunostaining of microglia and astrocytes in cortex. (A) Representative images of fluorescent co-immunostaining of microglia (Iba1, red) and astrocytes (GFAP, green) in the cortex of C57Bl/6J mice inoculated with 22L prions and normal age-matched controls (Norm). DAPI (blue) was used for staining of nuclei. (B) Magnified merged images of cortex of 22L-infected and normal age-matched C57Bl/6J mice.

Figure S3. Histopathological analysis of SSLOW-Mo-infected mouse brains. Low magnification images of deposition of PrP^{Sc} stained with SAF-84 antibody, and activated microglia and astrocytes stained for Iba1 and GFAP, respectively, in cortex, hippocampus and thalamus of C57Bl/6J mice inoculated with 22L prions. Scale bar = 500 μm.

Figure S4. Comparison of region-specific microglial and astrocytic response to 22L prion infection in individual animals. Quantification of Iba1 (left) and GFAP (right) immunofluorescence in thalamus (Th) and stratum oriens of the hippocampus (Hp s-or) of C57Bl/6J mice inoculated with 22L prions and normal age-matched controls (Norm). Each bar represents the mean integrated density for at least 30 regions of interest collected from 3 images. Error bars – Standard Error of measurements.

Figure S5. Analysis of gene expression by qRT-PCR. The ΔC_t of A1 markers Serping1 and Ggta1, A2 markers Tgm1 and S100a10, and proinflammatory marker Cxcl10 in thalamus (Tha), hippocampus (Hipp) and cortex (Cort) of C57Bl/6J mice inoculated with 22L prions and normal age-matched controls (N). The mean and standard deviation are shown (n=3 individual animals). Each symbol represents an individual mouse. GAPDH was used as a housekeeping gene. Statistical significance (P) was calculated by Student's unpaired t-test and indicated as * for $p<0.05$; ** for $p<0.01$; *** for $p<0.001$; **** for $p<0.0001$; ns for non-significant.
