# Supplementary figures and images for: Region-Specific Response of Astrocytes to Prion Infection
Source: Front Neurosci. 2019 Oct 9;13:1048. doi: 10.3389/fnins.2019.01048 (PMC6794343; doi:10.3389/fnins.2019.01048)

Fig. S1

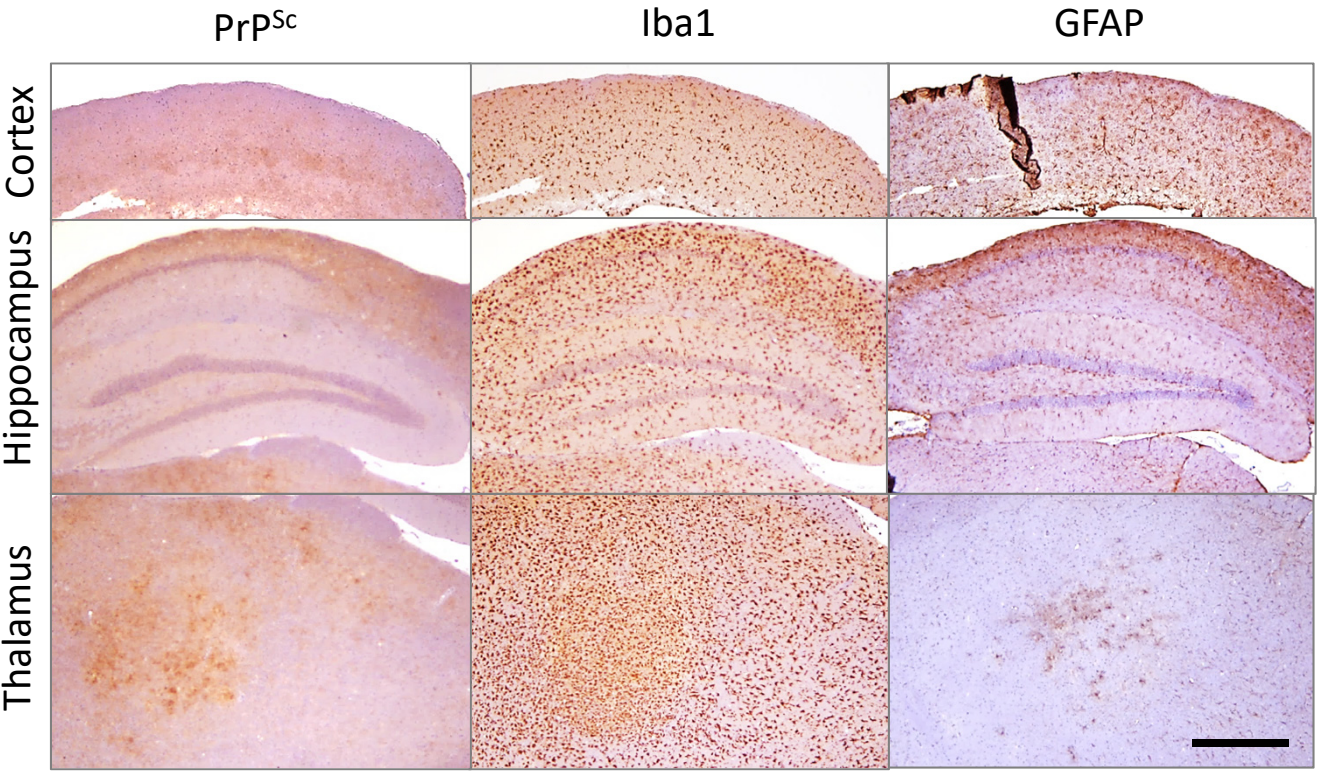

Fig. S2

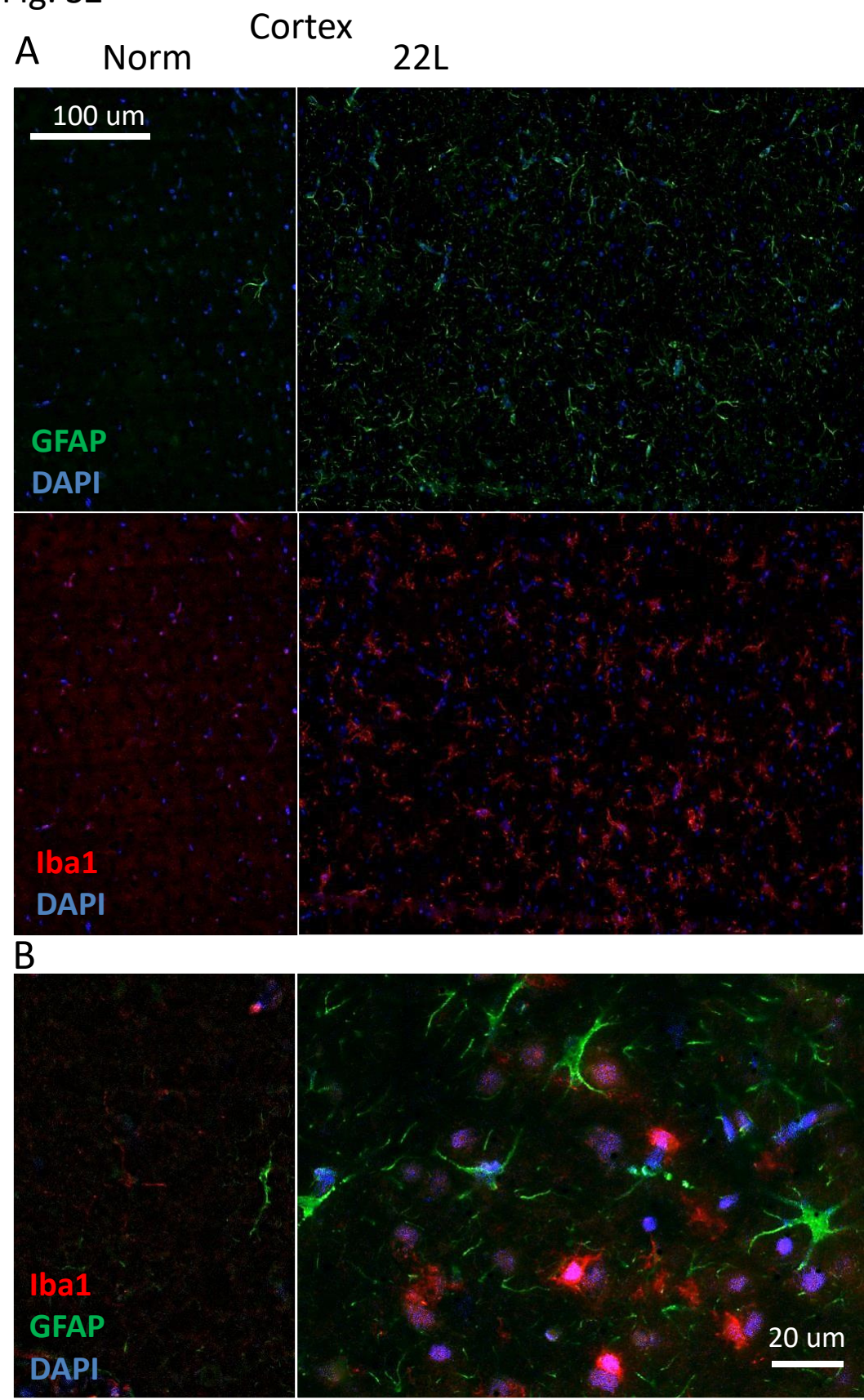

Fig. S3

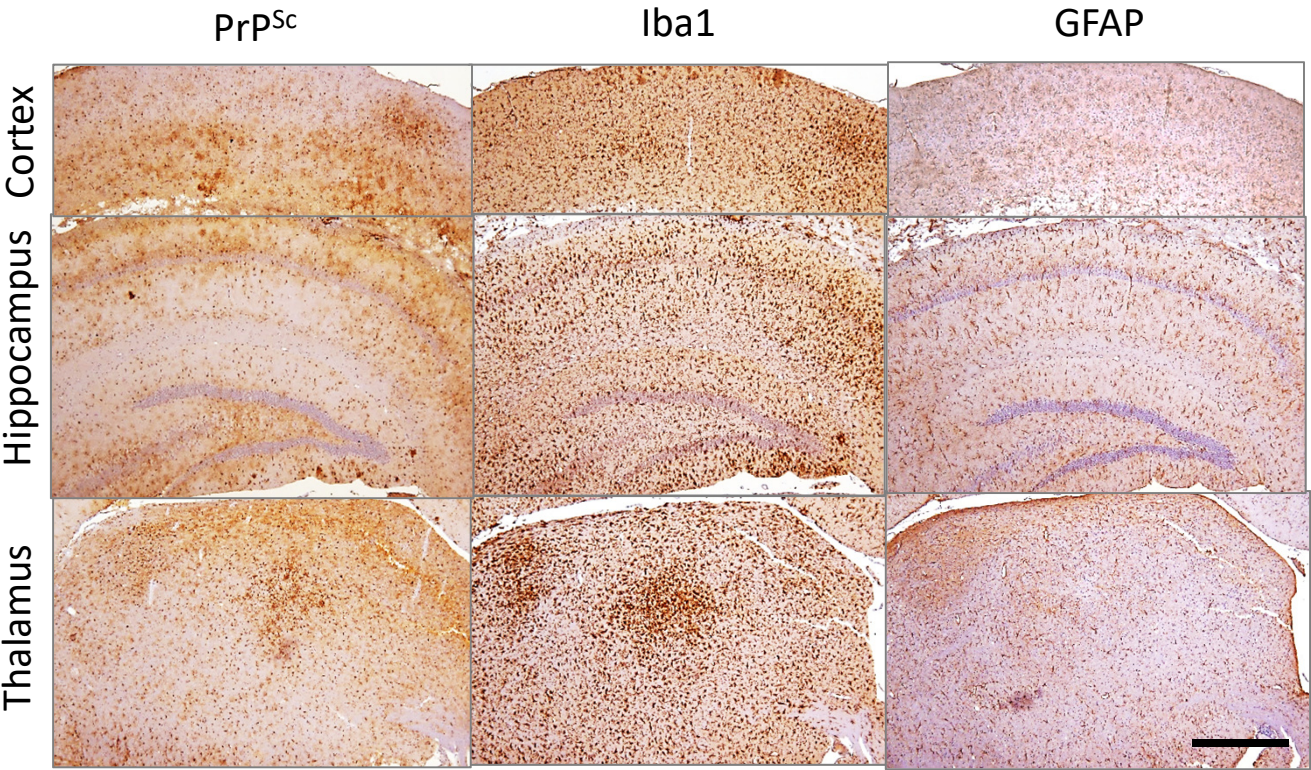

Fig. S4

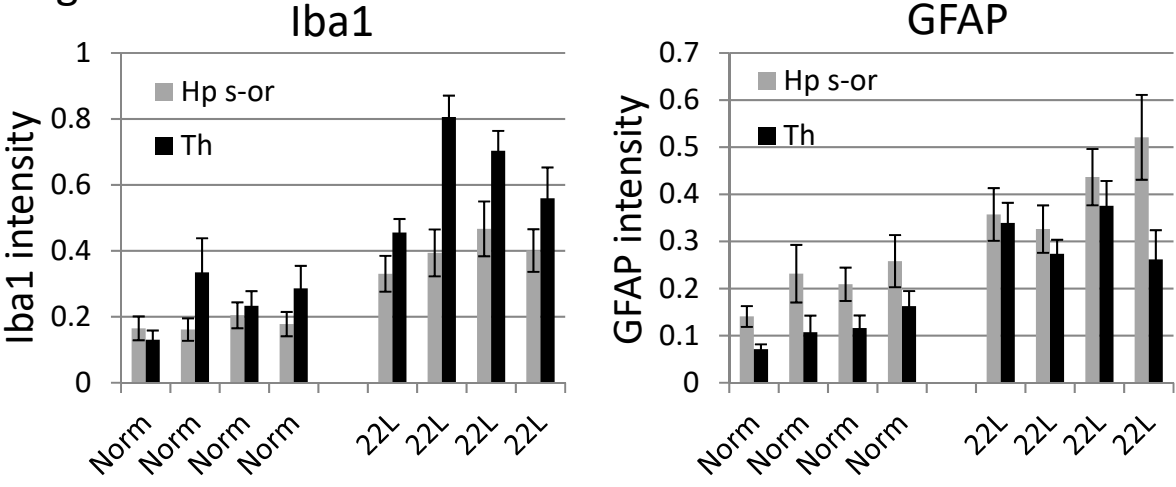

Fig. S5

A1 markers

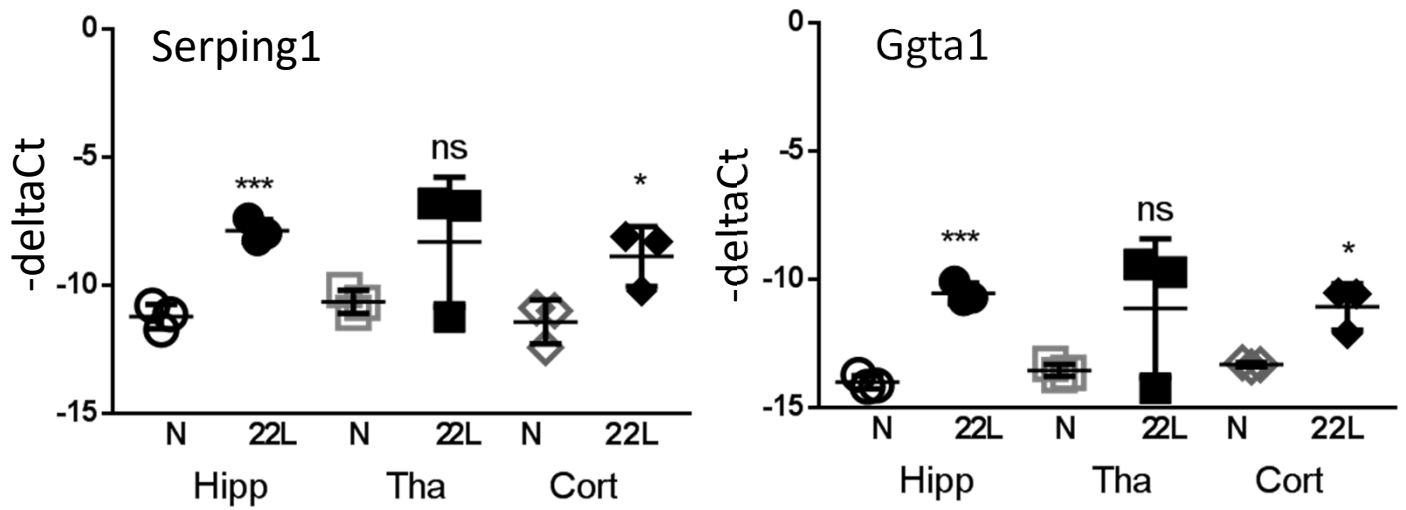

A2 markers

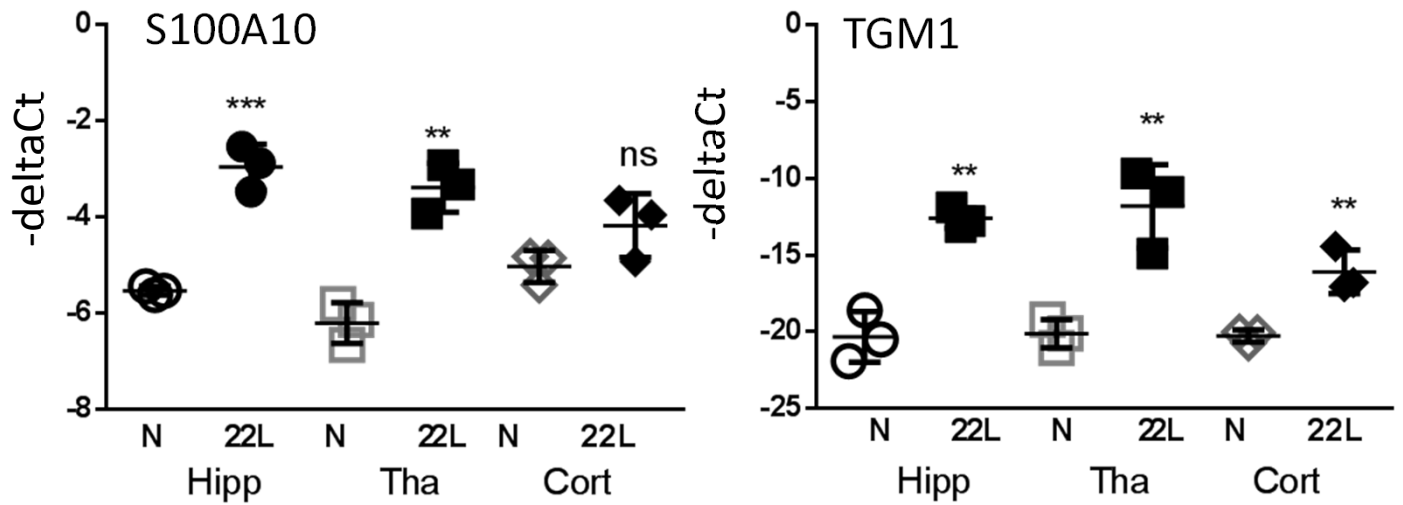

proinflammatory

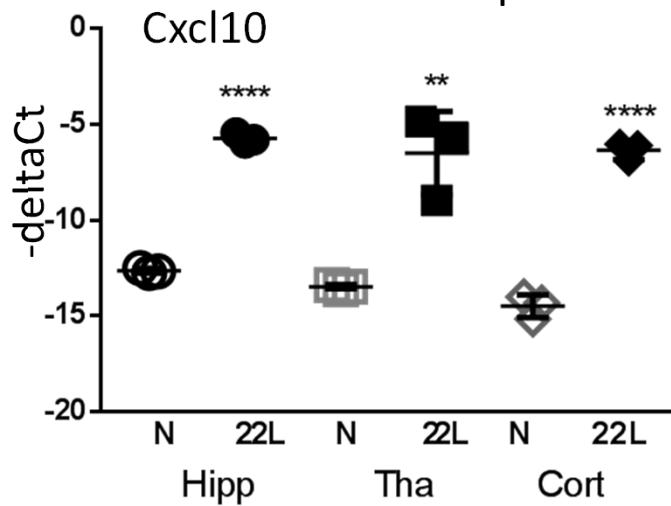

Supplement: Supplementary file 2 [file Data_Sheet_2.PDF]
